# A Diagnostic Challenge: Disseminated Herpes Simplex Virus Type 2 Infection Presenting in Stages and Mimicking Sepsis and Biliary Disease Across Two Healthcare Systems

**DOI:** 10.7759/cureus.112945

**Published:** 2026-07-19

**Authors:** Nicole Fontanet-Gomez, Antoine Khoudari, Maria Cekan

**Affiliations:** 1 Internal Medicine, BayCare Health System, Tampa, USA; 2 Family Medicine, BayCare Health System, Tampa, USA

**Keywords:** disseminated viral infection, elevated liver enzyme, hsv-2, periportal edema, sjögren’s syndrome

## Abstract

Disseminated herpes simplex virus type 2 (HSV-2) infection in adults is rare but can result in severe complications including hepatitis, encephalitis, and sepsis-like illness. Early symptoms are often nonspecific and may mimic common conditions, leading to delayed diagnosis and treatment. We present the case of a 74-year-old woman with Sjögren's syndrome who initially developed left upper eyelid erythema and swelling, followed by a viral-like prodrome with fever and myalgia, and subsequent urinary symptoms prior to traveling abroad. She was hospitalized in Spain with fever, dysuria, and transaminitis. Abdominal ultrasound demonstrated gallbladder wall thickening and free fluid without biliary obstruction, and she was empirically treated with broad-spectrum antibiotics and corticosteroids for presumed urinary tract infection and biliary disease. A language barrier complicated communication and clinical evaluation. After approximately one week of persistent symptoms, she returned to the United States and was admitted to our institution, where she was found to have culture-negative sepsis, hyponatremia, encephalopathy, anasarca, and persistent transaminitis. Extensive bacterial and fungal infectious workup was negative. HSV-2 polymerase chain reaction (PCR) was positive, confirming disseminated HSV-2 infection. During the latter part of hospitalization, she developed a small cluster of vesicular lesions. The patient improved with intravenous acyclovir followed by oral valacyclovir. Diagnostic complexity was compounded by care across different healthcare systems and a language barrier. This case highlights the staged progression of disseminated HSV-2 infection, from localized mucocutaneous involvement through a systemic viral prodrome to organ-threatening disease, and underscores the importance of considering disseminated HSV-2 in patients presenting with culture-negative sepsis and transaminitis. Early recognition and prompt antiviral therapy are essential to reduce morbidity and mortality.

## Introduction

Herpes simplex virus type 2 (HSV-2) is a common lifelong viral infection and the primary cause of genital herpes. The most recent WHO-commissioned estimates (2020 data) found that 519.5 million people aged 15-49 years had prevalent HSV-2 infection, representing 13.3% of the global population in that age group [1]. Despite this high seroprevalence, disseminated HSV-2 infection in adults remains rare and carries high mortality. A retrospective study of HSV viremia in hospitalized adults found the most common clinical findings were hepatitis (62%), fever (54%), CNS alterations (46%), skin lesions (38%), abdominal pain (31%), and sepsis (31%), with mortality in 6 of 10 immunocompromised and 1 of 3 immunocompetent patients [2]. The CDC STI Treatment Guidelines recommend intravenous acyclovir (5-10 mg/kg every 8 hours) for disseminated infection, pneumonitis, hepatitis, or CNS complications [3].

The diagnosis of disseminated HSV infection is often challenging because it typically evolves in stages, with early nonspecific symptoms that may mimic common conditions such as viral illness, urinary tract infection, biliary disease, or bacterial sepsis. Characteristic vesicular lesions may appear late in the disease course or be absent entirely, further delaying diagnosis and treatment [4]. Hepatic involvement may present with marked transaminitis and imaging findings such as gallbladder wall edema, pericholecystic fluid, and ascites, mimicking biliary disease in the absence of biliary obstruction. Gallbladder wall thickening (>3 mm) has been reported in 68% of patients with acute viral hepatitis and reflects hepatic dysfunction rather than primary biliary pathology [5]. HSV hepatitis typically presents as anicteric hepatitis with elevated transaminases, fever, flu-like symptoms, abdominal pain, leukopenia, or thrombocytopenia, and herpetic mucocutaneous lesions are detected in less than half of patients [6].

HSV-2 is also the predominant cause of Elsberg syndrome, a rare lumbosacral meningoradiculitis. A Danish nationwide cohort study found that all 28 patients with viral lumbosacral radiculitis had urinary retention, with 61% requiring catheterization; HSV-2 was identified in 68% of cases [7]. Urinary retention can also occur during primary genital HSV-2 infection (reported in up to 26% of incident HSV-2 cases) and may precede or occur without visible genital lesions, potentially leading to misdiagnosis as urinary tract infection [8].

While disseminated HSV is most commonly reported in immunocompromised individuals, including transplant recipients, patients with HIV, and those receiving cytotoxic chemotherapy, it can also occur in patients with autoimmune conditions and subtle immune dysregulation, even in the absence of immunosuppressive therapy. Sjögren's syndrome (SjS) is characterized by chronic immune dysregulation involving both innate and adaptive immunity, with B-cell hyperactivation, hypergammaglobulinemia, and disrupted interferon signaling pathways [9]. Separately, a French nationwide study of 25,661 patients with primary SjS demonstrated a significantly increased risk of hospitalization for infections, including a 3.3-fold increased risk for herpes zoster (aHR 3.32, 95% CI 1.78-6.18), even after adjusting for immunosuppressive therapy use [10]. This elevated susceptibility to herpesvirus reactivation suggests that the immune dysregulation intrinsic to SjS, including impaired interferon signaling and altered innate and adaptive immune responses, may reduce antiviral immune surveillance, increasing susceptibility to HSV reactivation and severe disseminated infection even in the absence of immunosuppressive therapy.

Furthermore, the keratoconjunctivitis sicca that defines SjS creates a compromised ocular surface with disrupted epithelial barrier function and chronic inflammation, which may facilitate viral entry and local reactivation [11]. Innate immune sensor dysfunction in the setting of systemic autoimmune disease further contributes to impaired mucosal defense [12]. HSV-2 blepharokeratoconjunctivitis is uncommon (1.4% of laboratory-proven HSV ocular infections in one series), and notably, classic dendritic lesions were present in only 22% of cases, while systemic immune disorders were identified in 22% of affected patients [13].

This case highlights the importance of considering disseminated HSV-2 infection in patients with autoimmune conditions presenting with culture-negative sepsis and transaminitis, even in the absence of immunosuppressive therapy.

## Case presentation

A 74-year-old woman with a history of SjS, who was not receiving immunosuppressive therapy, presented with a progressive multisystem illness that evolved over several weeks. Her medication regimen included levothyroxine, rosuvastatin, clonazepam, dextroamphetamine-amphetamine, and denosumab for osteoporosis, without any immunosuppressive agents. Her illness progressed in recognizable stages. It began with left upper eyelid erythema and swelling that was diagnosed as anterior blepharitis. Three days later, while traveling to Spain, she developed a viral-like prodrome characterized by fever, myalgia, malaise, and urinary symptoms, which subsequently progressed to hepatitis with marked transaminitis, encephalopathy, hyponatremia, and a sepsis-like syndrome. Her clinical course was further complicated by hospitalization in a different country, where a language barrier limited communication and continuity of care, contributing to delayed diagnosis and treatment.

On Day 1, she developed ocular symptoms including eye irritation, swelling, and visual discomfort. Over the following days, she developed malaise, fatigue, and subjective fevers. On Day 7, she developed dysuria and urinary discomfort. Given her planned departure to Spain the following day, she sought evaluation through a telehealth visit and was empirically prescribed nitrofurantoin for presumed urinary tract infection coverage. No urinalysis, urine culture, or laboratory testing was performed before initiation of treatment.

On Day 10, while traveling in Spain, she developed persistent fevers, worsening fatigue, and generalized weakness requiring hospitalization. Laboratory evaluation demonstrated marked hepatocellular injury with AST 357 U/L and ALT 529 U/L, hyponatremia (Na 128 mmol/L), and elevated inflammatory markers (CRP 24.1 mg/L) (Table 1). Blood and urine cultures were reportedly negative. She received empiric treatment with antibiotics and corticosteroids; however, due to persistent symptoms and difficulty communicating because of a language barrier, she left the hospital against medical advice and returned to the United States.

**Table 1:** Chronological laboratory trends during the patient's clinical course. Abbreviations: AST, aspartate aminotransferase; ALT, alanine aminotransferase; CRP, C-reactive protein. Reference ranges: sodium 136-145 mmol/L; AST 5-34 U/L; ALT 0-55 U/L; alkaline phosphatase 40-150 U/L; albumin 3.2-5.0 g/dL; CRP 0.00-0.50 mg/dL.

Time Point	Sodium (mmol/L)	AST (U/L)	ALT (U/L)	Alkaline Phosphatase (U/L)	Albumin (g/dL)	CRP (mg/dL)
Day 10 (Spain hospitalization)	128	357	529	Not analyzed	2.1	24.1
Day 15 (Admission)	127	80	192	195	2.5	Not analyzed
Day 16	130	44	137	238	2.6	Not analyzed
Day 17	133	48	119	278	2.7	3.91
Day 18	131	68	116	321	2.7	Not analyzed
Day 21	135	48	86	326	3.6	Not analyzed
Day 28 (Outpatient follow-up)	136	32	38	204	3.5	0.79
Day 45 (Late follow-up)	139	32	29	128	3.9	Not analyzed

On Day 15, the patient presented directly from the airport to our emergency department for evaluation of persistent fever, dizziness, flank pain, abdominal pain, generalized weakness, and altered mental status. Neurologic examination was notable for confusion and altered mental status without focal neurologic deficits. Initial laboratory evaluation demonstrated leukocytosis, severe hyponatremia, elevated liver enzymes, cholestatic liver enzyme abnormalities, hypoalbuminemia, and elevated procalcitonin (Table 1). Computed tomography of the abdomen and pelvis demonstrated periportal edema, gallbladder wall edema, small bilateral pleural effusions, and low-volume abdominal ascites. Chest radiography demonstrated bibasilar volume loss versus infiltrates and small pleural effusions. A non-contrast CT of the head demonstrated no acute intracranial abnormalities.

**Figure 1:**
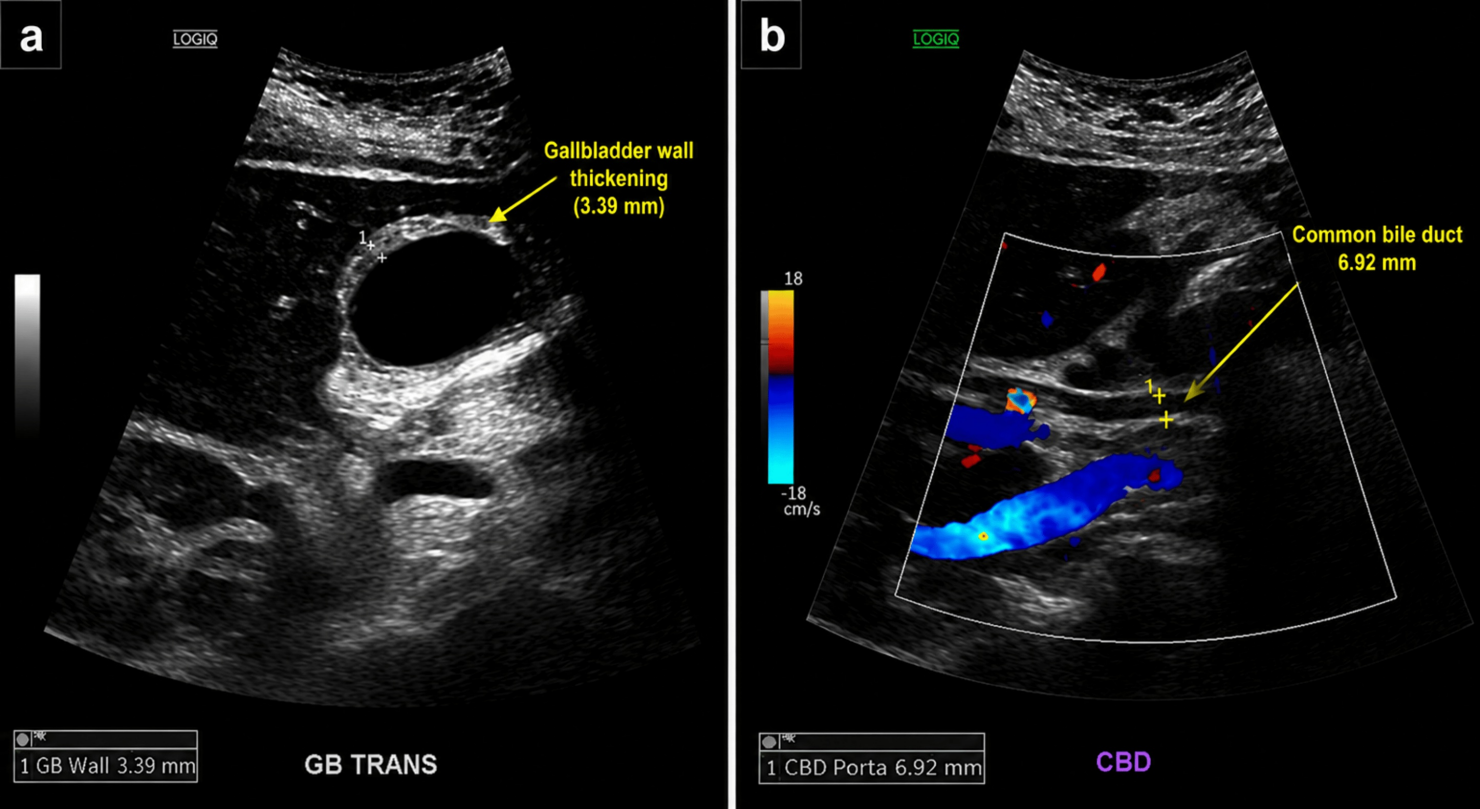
Abdominal ultrasound obtained during hospitalization. (A) Transverse gallbladder ultrasound demonstrating mild gallbladder wall thickening measuring 3.39 mm without visualized cholelithiasis. (B) Color Doppler ultrasound demonstrating a mildly prominent common bile duct measuring 6.92 mm without definite obstructive biliary dilation.

Abdominal ultrasound obtained during hospitalization demonstrated mild gallbladder wall thickening without cholelithiasis and a mildly prominent common bile duct without definite obstructive biliary dilation (Figure 1).

Despite persistent fevers and concern for an infectious process, blood cultures and urine cultures remained negative throughout hospitalization. Additional infectious workup, including respiratory viral panel, hepatitis A, B, and C serologies, HIV testing, and COVID-19 testing, was negative. Urinalysis demonstrated trace protein, trace blood, leukocyte esterase positivity, and 11-15 white blood cells per high-power field; however, urine cultures remained negative. Autoimmune evaluation revealed positive antinuclear antibodies (ANA) and positive SS-A antibodies, consistent with her known history of SjS.

**Figure 2:**
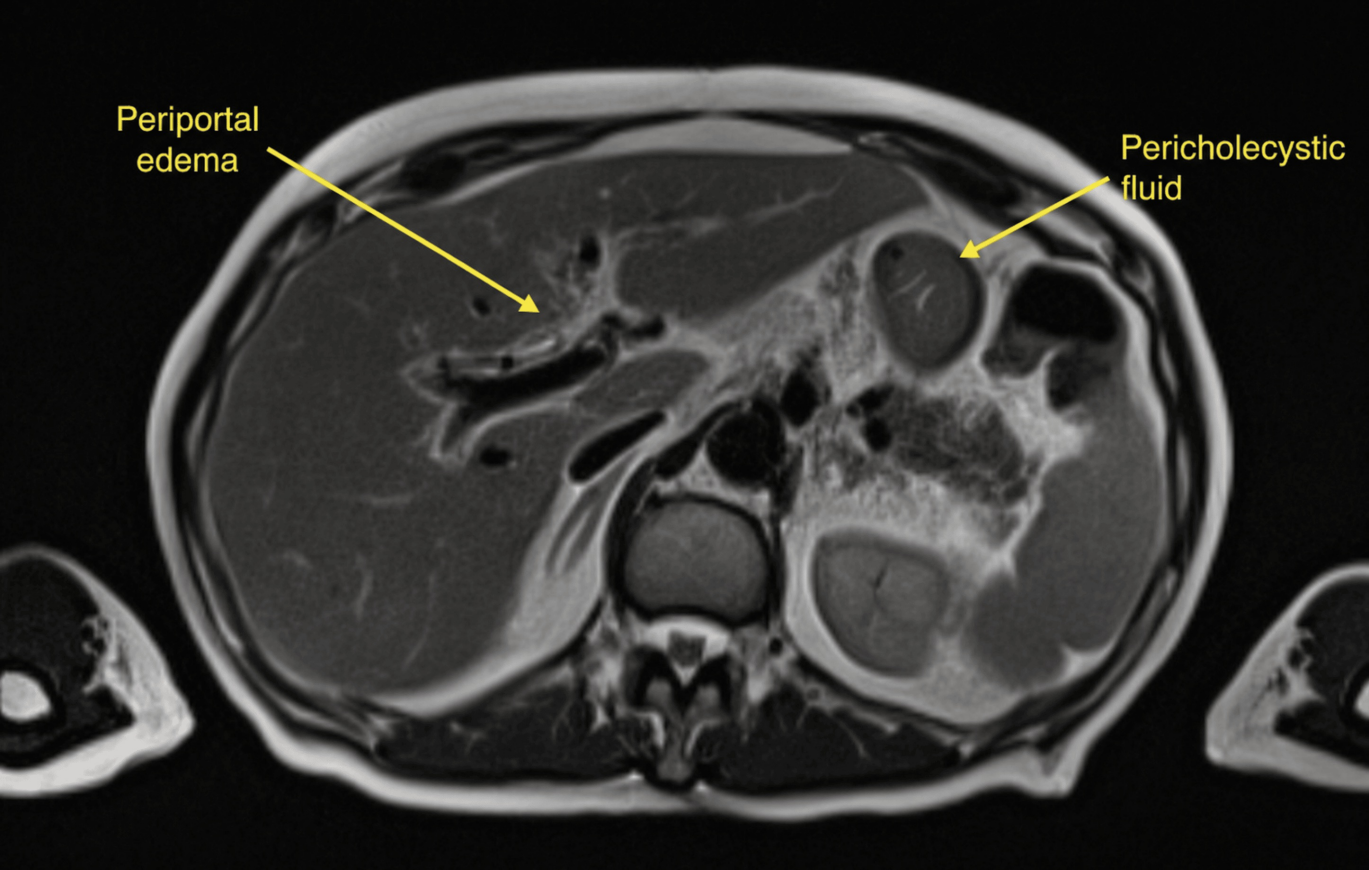
MRI/MRCP during acute illness. Axial T2-weighted MRI demonstrating hepatic heterogeneity with periportal edema and pericholecystic fluid without biliary ductal dilation or choledocholithiasis. These findings were initially concerning for biliary pathology but were ultimately attributed to disseminated HSV-2 infection with hepatic involvement. *Abbreviations*: MRI, magnetic resonance imaging; MRCP, magnetic resonance cholangiopancreatography; HSV-2, herpes simplex virus type 2.

On Day 17, magnetic resonance imaging (MRI) of the abdomen with magnetic resonance cholangiopancreatography (MRCP) demonstrated hepatic heterogeneity with periportal edema, pericholecystic fluid, and mild ascites without evidence of biliary ductal dilation or choledocholithiasis (Figure 2). Although initially concerning for hepatobiliary pathology, the absence of biliary obstruction suggested a systemic inflammatory or infectious process rather than primary biliary disease.

During hospitalization, the patient developed encephalopathy characterized by confusion and altered mental status, persistent fevers, worsening fatigue, anasarca, persistent transaminitis, and ongoing hyponatremia. Given the combination of persistent fever, systemic inflammatory illness, hepatitis, encephalopathy, and unrevealing bacterial cultures, a viral etiology was suspected. On Day 17, further diagnostic evaluation demonstrated a positive HSV-2 plasma polymerase chain reaction (PCR) performed using a qualitative real-time PCR assay targeting the HSV DNA polymerase gene, confirming disseminated HSV-2 infection.

Following confirmation, the patient was started on intravenous acyclovir. During the latter part of her hospitalization, she developed a small cluster of two to three vesicular lesions over the right buttock, which had nearly resolved by the time of outpatient follow-up. She subsequently demonstrated progressive clinical improvement, including resolution of fevers, normalization of mental status, improvement in serum sodium levels, and downtrending liver enzymes. She was later transitioned to oral valacyclovir to complete antiviral therapy.

At outpatient follow-up, the patient reported marked symptomatic improvement with resolution of fevers, weakness, and encephalopathy. Follow-up laboratory testing demonstrated normalization of serum sodium and substantial improvement in liver function tests. Repeat laboratory evaluation on Day 28 demonstrated near-complete normalization of transaminases with continued improvement in alkaline phosphatase levels (Table 1).

**Figure 3:**
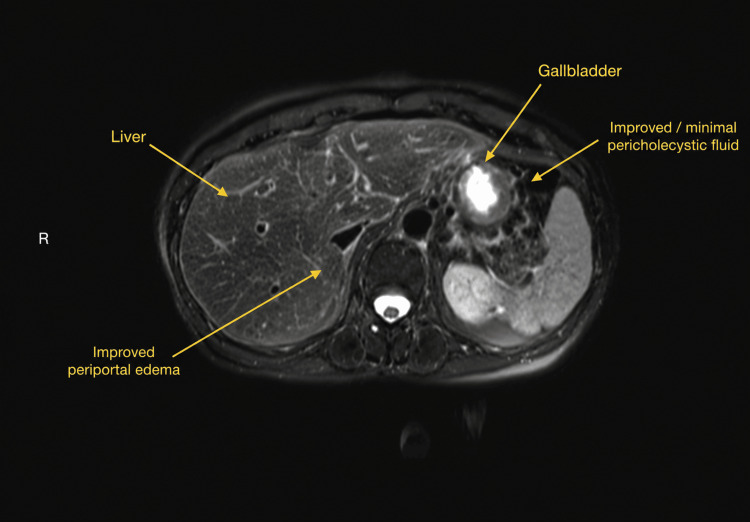
Follow-up axial T2-weighted fat-suppressed MRI demonstrating marked interval resolution of periportal edema and minimal residual pericholecystic fluid following antiviral therapy for disseminated HSV-2 infection. *Abbreviations:* MRI, magnetic resonance imaging; HSV-2, herpes simplex virus type 2.

A follow-up MRI obtained on Day 45 demonstrated marked interval improvement in periportal edema and pericholecystic inflammatory changes, with only minimal residual abnormalities remaining (Figure 3).

## Discussion

Disseminated HSV-2 infection in adults is rare but carries substantial morbidity and mortality, particularly among elderly and immunocompromised patients, and can involve the liver, central nervous system, and genitourinary tract simultaneously. HSV hepatitis alone carries a mortality rate of up to 90% without specific therapy, making it the most lethal complication of disseminated disease [14], and a retrospective study of HSV viremia in hospitalized adults corroborates this severity, with death occurring in 6 of 10 immunocompromised patients and 1 of 3 immunocompetent patients [2]. Because outcomes are so tightly linked to how early therapy begins, understanding why this diagnosis is so often missed is as clinically important as recognizing the disease itself.

Why the diagnosis is missed: staged and nonspecific presentation

The central diagnostic challenge in this case was the staged, seemingly unrelated progression of symptoms: ocular findings, followed by a flu-like viremic phase, then urinary symptoms, and only later hepatitis, encephalopathy, hyponatremia, and a sepsis-like picture. This sequential unmasking is a recognized but underappreciated pattern in disseminated HSV infection, and it is compounded by the fact that characteristic vesicular lesions, the finding clinicians rely on to suspect HSV, are absent or appear late in the majority of cases; herpetic mucocutaneous lesions are detected in fewer than half of patients with HSV hepatitis [6]. In this patient, that diagnostic delay was further amplified by international travel and a language barrier, which limited history-taking and continuity of care abroad, a systems-level factor that, layered onto an already atypical presentation, meant multiple opportunities for early suspicion were lost before the unifying diagnosis became apparent.

Genitourinary involvement

The patient's early urinary symptoms likely reflect a genitourinary manifestation of HSV-2 rather than an incidental urinary tract infection. While HSV-associated lumbosacral radiculitis (Elsberg syndrome) is a recognized cause of urinary dysfunction in this setting, a Danish nationwide cohort found urinary retention in all 28 patients with viral lumbosacral radiculitis, with 61% requiring catheterization and HSV-2 identified in 68% of cases [7]; our patient did not meet sufficient criteria for this diagnosis based on the available clinical picture. However, this determination is limited by the absence of confirmatory testing: neither cerebrospinal fluid analysis nor lumbar spine MRI was performed, so Elsberg syndrome cannot be formally excluded and remains a diagnostic possibility rather than a ruled-out entity. More broadly, urinary retention is reported in up to 26% of incident primary genital HSV-2 infections and can precede or occur entirely without visible genital lesions [8], which likely explains why this symptom was initially misattributed rather than recognized as a herald of systemic viral spread.

Hepatic involvement

Hepatic disease was the most severe organ manifestation in this case and is a well-described feature of disseminated HSV, typically presenting, as it did here, as anicteric hepatitis with marked transaminitis, thrombocytopenia, and leukopenia rather than the cholestatic picture more typical of other hepatitides [6]. Imaging findings can further mislead: gallbladder wall thickening greater than 3 mm is present in 68% of patients with acute viral hepatitis and can closely mimic acalculous cholecystitis or primary biliary pathology, when in fact it reflects hepatic inflammation and periportal edema rather than intrinsic gallbladder disease [5]. Recognizing this imaging pattern as a consequence of viral hepatitis, rather than a separate biliary process, is an important step in preventing unnecessary biliary work-up and further delay.

Central nervous system involvement and hyponatremia

The patient's encephalopathy and hyponatremia raise the possibility of central nervous system involvement. This association is notable in the literature: among patients with acute CNS infections, hyponatremia affects 39% overall, and HSV specifically carries the highest odds of hyponatremia among causative organisms (OR 3.25, 95% CI 1.13-7.87) [15], an effect thought to be mediated via the syndrome of inappropriate antidiuretic hormone secretion (SIADH), a well-established consequence of CNS pathology more broadly [16]. However, this could not be confirmed in our patient, as cerebrospinal fluid analysis was not performed, and so direct HSV invasion of the CNS remains presumptive rather than established. Alternative explanations, including hepatic encephalopathy given the degree of transaminitis, or non-osmotic ADH release driven by critical illness and sepsis physiology more broadly, cannot be excluded. Given this association and in the absence of CSF data to confirm or refute CNS involvement in our patient, HSV encephalitis should remain a differential consideration in similar future cases, ideally prompting CSF analysis, including HSV PCR, where clinically feasible, to establish or exclude this diagnosis definitively.

Host susceptibility: Sjögren's syndrome (SjS) and corticosteroid exposure

Two converging host factors likely permitted viral dissemination in this patient. First, her underlying SjS, characterized by B-cell hyperactivation, hypergammaglobulinemia, and disrupted interferon signaling [9], independently predisposes to severe herpesvirus reactivation: a French nationwide study of 25,661 patients with primary SjS found a 3.3-fold increased risk of hospitalization for herpes zoster (aHR 3.32, 95% CI 1.78-6.18), an association that persisted after adjusting for immunosuppressive therapy [10]. This indicates that the immune dysregulation intrinsic to SjS itself, separate from any treatment received for it, is sufficient to elevate the risk of severe viral disease.

Second, this intrinsic vulnerability was compounded by corticosteroids the patient received while hospitalized abroad, plausibly for a presumed non-viral inflammatory or infectious process before HSV was suspected. Corticosteroids activate the glucocorticoid receptor in a manner that directly stimulates HSV productive infection and viral gene expression, while stress-induced glucocorticoids independently suppress antiviral CD8+ T-cell responses, impairing viral clearance [17,18]. This combination of mechanisms, direct promotion of viral replication plus suppression of the cellular immunity needed to control it, has been implicated in prior reports of disseminated HSV-1 with multi-organ involvement (hepatitis, encephalitis, myocarditis) following corticosteroid treatment even in patients without major underlying immunosuppression [19]. Taken together, SjS likely established a baseline susceptibility to herpesvirus reactivation, and corticosteroid exposure then acted as the probable trigger for full dissemination.

Diagnostic and therapeutic implications

This case underscores the importance of considering viral, and specifically HSV, etiologies in patients presenting with sepsis of unclear source, transaminitis, encephalopathy, and negative bacterial cultures, particularly when an autoimmune history or recent corticosteroid exposure is present. Serum PCR for HSV DNA is the recommended confirmatory test, as all patients with HSV hepatitis have high viral DNA levels in serum [6]. Once disseminated HSV-2 was confirmed here, treatment with intravenous acyclovir followed by oral valacyclovir produced significant clinical and laboratory improvement, consistent with CDC STI Treatment Guidelines, which recommend intravenous acyclovir (5-10 mg/kg every 8 hours) for disseminated infection, pneumonitis, hepatitis, or CNS complications [3]. The stakes of early treatment are well established: mortality from HSV encephalitis was approximately 70% in the pre-antiviral era, falling to roughly 20% with acyclovir, though morbidity among survivors remains high [20]. HSV therefore remains one of the few causes of acute liver failure and encephalopathy that are both treatable and reversible when identified promptly, making early recognition, rather than any single confirmatory test, the true determinant of outcome in cases like this one.

## Conclusions

Disseminated HSV-2 infection is a rare but important cause of culture-negative sepsis, hepatitis, encephalopathy, and multisystem illness in adults. Because early manifestations may be nonspecific and mimic urinary, biliary, or bacterial disease, diagnosis is often delayed. This case demonstrates the staged progression of disseminated HSV-2 infection across multiple organ systems and healthcare settings. Clinicians should maintain a high index of suspicion for HSV infection in patients with unexplained sepsis, transaminitis, negative cultures, or new vesicular lesions, particularly in elderly or immunologically vulnerable individuals. Early recognition and prompt antiviral therapy may significantly reduce morbidity and improve outcomes.
